# Traditional Chinese medicine for restless legs syndrome: A study protocol for a network meta-analysis

**DOI:** 10.1097/MD.0000000000022831

**Published:** 2020-10-30

**Authors:** Liting Liu, Rongfang Xie, Ruiqi Wang, Chunhua Huang

**Affiliations:** a Jiangxi University of Traditional Chinese Medicine Nanchang; b Affiliated Hospital of Jiangxi University of Traditional Chinese Medicine, Nanchang, Jiangxi Province, China.

**Keywords:** traditional Chinese medicine, acupuncture, restless legs syndrome, network meta-analysis, protocol

## Abstract

**Background:**

Restless legs syndrome (RLS) is a sensory motor disorder. It mainly manifests as indescribable pain in the lower limbs at night or at rest, and the symptoms are reduced after activity or beating, which seriously affects the patients sleep. Nowadays, a large number of randomized controlled clinical studies have shown that Chinese medicine has the advantages of good curative effect and high safety in the treatment of RLS. However, due to the various treatment methods of Chinese medicine, its relative effectiveness and safety have not been verified. Therefore, this study will use a network meta-analysis method to verify the effectiveness and safety of different types of TCM therapies in the treatment of RLS.

**Methods:**

Computer retrieval was conducted in PubMed, Cochrane Library, Web of Science, Embase, SinoMed, CNKI, WanFang-database, VIP. The retrieval period was until September 9, 2020, and all randomized controlled trials of TCM treatment of RLS were collected. To avoid omissions, we will manually search relevant references and conference papers. According to the inclusion and exclusion criteria, we conduct quality assessment and risk assessment of all retrieved documents. Methodological quality assessment and risk of bias will be assessed using Cochrane bias risk tool. All data analysis will use Revman5.3, WinBUGS 1.4.3, and Stata14.2 software.

**Results:**

This study will directly or indirectly compare the effectiveness of different interventions on RLS outcome indicators, and rank the effectiveness. The main outcome indicators include total effective rate (total effective rate = rocovery + obvious effective + effective/total number of cases × 100%), International Restless Legs Syndrome Score Scale, secondary outcome indicators include visual analog scale, Pittsburgh sleep quality indicators and adverse events.

**Conclusion:**

Provide a basis for evidence-based medicine, and provide a basis for clinical researchers to choose more effective Chinese medicine treatment of RLS.

## 1 Introduction

Restless legs syndrome (RLS) is a clinically common sleep-related sensorimotor disorder, the main clinical manifestations are indescribable paresthesia and discomfort in the lower limbs at rest or night sleep, and a strong desire to move the lower limbs. The symptoms are relieved after the activity and reappear after stopping.^[1]^ RLS can be divided into 2 types: primary and secondary according to the presence or absence of primary disease. Primary may be genetically related, and secondary causes include spinocerebellar ataxia, Parkinsons disease, and iron deficiency anemia. Epidemiology shows that in the white adult population, the estimated range of prevalence is 7.2% to 11.5%. The prevalence of RLS increases with age, and women are more likely to be affected than men.^[2]^ Western medicine treatment of this disease includes symptomatic treatments such as iron supplementation, improvement of lower limb blood circulation, and analgesia, dopamine receptor agonists are the first choice for the treatment of RLS.^[3]^ However, some studies have shown that long-term high-dose dopamine agonists are prone to deterioration, often leading to treatment interruption.^[4]^ Therefore, we urgently need to explore more effective and safe treatment methods.

As the traditional medicine of China, traditional Chinese medicine has been passed down to this day because of its remarkable efficacy and high safety advantages. Traditional Chinese medicine has a history of thousands of years in China and has gradually been recognized by countries all over the world. Chinese medicine is believed to be effective in improving various diseases, including RLS. Traditional Chinese medicine believes that the etiology and pathogenesis of RLS are not single, so there are many corresponding treatment methods, such as traditional Chinese medicine, Chinese patent medicine, acupuncture, moxibustion, and massage. Studies have shown that acupuncture treatment of RLS can trigger the release of analgesic neuropeptides to relieve pain.^[5]^ Chinese herbal medicine Radix Paeoniae Alba is one of the most commonly used Chinese medicines for the treatment of RLS. It has the functions of relaxing blood vessels, improving distal vascular circulation, regulating and improving blood circulation in extremities.^[6]^ At present, some standard meta-analysis shows that TCM is definitely effective in the treatment of RLS,^[7,8]^ But all of these are simply a traditional Chinese medicine method compared to a simple Western medicine, and it is impossible to compare multiple Chinese medicine methods. As we know, there are many treatment methods in traditional Chinese medicine, and the advantages of treatment are different, so the choice of which treatment method to use has brought troubles to clinical operators. The network meta-analysis can make positive comparisons of various interventions. Therefore, we will use network meta-analysis to systematically compare the effectiveness and safety of different TCM interventions, and provide evidence-based medicine for clinical researchers.

## 2 Protocol registration

This system review program will strictly follow the system review and meta-analysis program (PRISMA-P) preferred report items for reporting.^[9]^ The system review program has been registered on the INPLASY website (the registration number is INPLASY202090041), If there are any adjustments during the entire study period, we will promptly revise and update the detailed information in the final report.

## 3 Methods

### 3.1 Inclusion and exclusion criteria

#### 3.1.1 Study type

RCTs based on different Chinese medicine treatments for restless legs syndrome, the language is limited to Chinese and English. Non-RCTs literature, such as conference reports, literature reviews will be excluded; Literatures on other folk remedies besides traditional Chinese medicine will be excluded; Literatures with unavailable data and full text will be excluded; Literatures that do not include the outcome indicators included in this study will be excluded. The most recent one shall be selected for repeated detection and repeated publication.

#### 3.1.2 Participants

Patients diagnosed as RLS in accordance with the internationally recognized diagnostic criteria have clear curative effect standards, regardless of age, race, gender, and source of cases. However, the following patients will be excluded:

1. Patients who are unwilling to receive TCM treatment,
2. Patients with serious cardiovascular and cerebrovascular diseases and mental illnesses.
3. Pregnant or lactating women.

#### 3.1.3 Interventions

The experimental group only uses traditional Chinese medicine, such as traditional Chinese medicine, proprietary Chinese medicine, acupuncture, moxibustion, and massage. The control group uses Western medicine alone. Both the experimental group and the control group can cooperate with conventional medical treatment.

#### 3.1.4 Outcome indicators

The included outcome indicators include 1 or more of the following: The main outcome indicators include total effective rate (total effective rate = rocovery + obvious effective + effective/total number of cases × 100%), International Restless Legs Syndrome Score Scale, secondary outcome indicators include visual analog scale, Pittsburgh sleep quality indicators And adverse events.^[10–12]^

### 3.2 Data sources and search strategies

**Figure 1:**
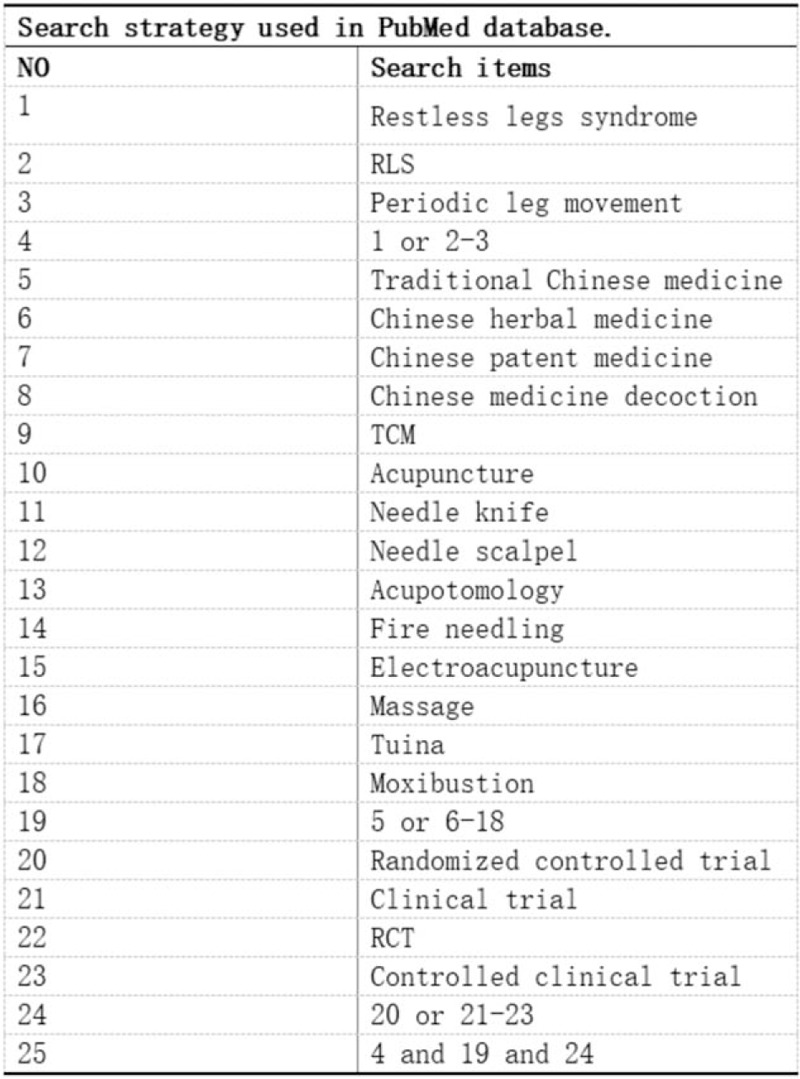
Search strategy used in PubMed database.

Computer retrieval was conducted in PubMed, Cochrane Library, Web of Science, Embase, SinoMed, CNKI, WanFang-database, VIP. The retrieval period was until September 9, 2020, The search terms are:“traditional Chinese medicine”, “Chinese herbal medicine”, “ Chinese patent medicine”, “Chinese medicine decoction” “acupuncture”, “moxibustion”, “massage”, “Restless legs syndrome”. The search strategy is to combine search terms with subject words and free words. The data retrieval strategy is shown in Figure 1.

### 3.3 Selection of studies and data extraction

The 2 evaluators (LTL and RFX) first screened independently according to the literature inclusion and exclusion criteria, and then cross-checked. If there is a difference, the third reviewer can make a decision. Establish a document information extraction table in EXCEL, and extract information including: author, publication time, number of cases, allocation method, intervention measures, treatment course, and outcome indicators, etc.

### 3.4 Risk assessment of bias

Refer to the guidelines of the Cochrane Handbook for Systematic Reviews of Interventions to evaluate the risk of bias in the literature finally included in this study. The evaluation criteria include 7 items: selections bias, performance bias, detect bias, attrition bias, reporting bias, and other bias. The evaluation results are evaluated in terms of “high risk”, “low risk” and “unclear risk”.^[13,14]^

### 3.5 Statistical analysis

Using Revman 5.3 software for bias assessment and standard Meta analysis, the outcome indicators were the ratio Ratio (or) of count data, the mean difference (MD) of measurement data, and the 95% confidence interval (95% CI) for the effect. Using WinBUGS 1.4.3 and Stata 14.2 for network meta analysis,^[15,16]^ In WinBUGS software, use the Markov Chain Monte Carlo (MCMC) method for Bayesian network Meta analysis, The simulation is carried out through 4 chains, the number of iterations is set to 50,000, among which the first 20,000 times are annealed to eliminate the influence of the initial value, and the step size is set to 10.^[17]^ At the same time, the potential scale reduction factor(potential scale reduction factor, PSRF) is used to evaluate the convergence of the results, When the PRSF is close to or equal to 1.00 (1.00 ≤ PSRF ≤ 1,05), it indicates that the results have good convergence and the obtained results are highly reliable.^[18]^ At the same time, Stata software is used to calculate the SUCRA (surface under the cumulative ranking curves, SUCRA) value and the area under the SUCRA curve in order to rank the efficacy of various interventions. The value range is 0 to 100. The larger the value and the larger the area under the curve, the better the effect of the intervention.

### 3.6 Assessment of inconsistency

Since this study involves many intervention measures, in the evidence network of each outcome indicator, the closed loop formed by the research with direct evidence and indirect evidence needs to be tested for inconsistency through Stata software. Calculate the inconsistency factor (IF), and judge whether there is inconsistency through the IF value and the P value.^[19]^ If IF is close to 0, 95%CI starts at 0, and *P* > .05, it is considered that the results of direct comparison and indirect comparison are consistent. At the same time, the node-split model is used to determine whether each node has local inconsistencies.^[20]^ If *P* > .05, the consistency model is adopted, otherwise, the inconsistency model is adopted. For the results obtained from the consistency model analysis, the stability of the results can be checked through the inconsistency model, when the inconsistency factors include 0 and the inconsistency standard deviation includes 1, and the random standard deviation of the consistent effect model is approximately equal to the standard deviation of the inconsistency model, the consistency model results are more stable and reliable .^[21,22]^

### 3.7 Heterogeneity, subgroup analysis, sensitivity analysis

The heterogeneity between trials is quantified by *I*^2^ and *P* values.^[23]^ For test results with obvious heterogeneity, the source of the heterogeneity should be analyzed. According to the different sources of heterogeneity, subgroup analysis can be carried out, such as treatment time, course of disease, underlying disease, race, gender, age, etc. If there is no clear source of heterogeneity, only descriptive analysis can be performed. The purpose of sensitivity analysis is to eliminate low-quality studies and different statistical models.^[24]^ Observe the heterogeneity of different experiments, observe whether the combined results change after different treatments, and analyze the strength, reliability and stability of the results.

### 3.8 Assessment of publication bias

If the outcome indicators include in study ≥10, We will use a funnel chart to assess publication bias.^[25]^ If the funnel chart appears asymmetry or the distribution is different, it indicates publication bias or small sample effect.

### 3.9 Ethics and dissemination

Since this is a protocol for systematic reviews and network meta-analysis, all data in this study comes from published studies and does not involve patients, so ethical approval is not required. The results of this research will be distributed to peer-reviewed journals and published in relevant conferences.

## 4 Discussion

In recent years, with the continuous development and improvement of the TCM career, more and more clinical randomized controlled trials have shown that TCM treatment of RLS has the characteristics of significant efficacy and low side effects, indicating that TCM treatment of RLS has accumulated rich experience and achieved certain results. Standard meta-analysis can only make a single comparison, while network meta-analysis can compare multiple interventions in pairs. We will use the method of network meta-analysis to compare several different traditional Chinese medicine interventions to get a ranking of effectiveness and safety, so as to provide evidence-based medicine for clinical decision-makers.

## Author contributions

**Conceptualization:** Liting Liu.

**Methodology:** Liting Liu, Rongfang Xie, Chunhua Huang.

**Project administration:** Ruiqi Wang

**Software:** Rongfang Xie

**Supervision:** Chunhua Huang.

**Writing – original draft:** Liting Liu.

**Writing – review & editing:** Chunhua Huang, Liting Liu.

## References

[1] Frauscher B Restless Legs Syndrome. Eur J Neurol 2019;12:272.

[2] Ghorayeb I Tison F Restless legs syndrome epidemiology. Presse Med 2010;39:564–70. 20334990 10.1016/j.lpm.2009.08.006

[3] SA V AR P Birgit H Reply to: Safety of dopamine agonists for treating restless legs syndrome. Movement Disord 2019;34:150–1. 10.1002/mds.27571 30653723

[4] Winkelman JW 0793 Nearly 25% of Restless Legs Syndrome (RLS) Patients nationally treated with dopamine agonists are taking higher doses than the maximum recommended by FDA and expert guidelines. Sleep 2020;Supplement_1: A302.

[5] Raissi GR Forogh B Ahadi T Evaluation of acupuncture in the treatment of restless legs syndrome: a randomized controlled trial. J Acupunct Meridian Stud 2017;10:346–50. 29078970 10.1016/j.jams.2017.08.004

[6] Yan X Wang WD Walters AS Traditional Chinese medicine herbal preparations in restless legs syndrome (RLS) treatment: a review and probable first description of RLS in 1529. Sleep Med Rev 2012;16:10.1016/j.smrv.2012.01.003 22459934

[7] Zhou R Li KS Zhuang LX Cumulative Meta-analysis of the efficacy of acupuncture in the treatment of restless legs syndrome. Tianjin Tradit Chin Med 2019;36:579–83. In Chinese.

[8] Wang S Zhao R A systematic review of the efficacy of acupuncture in the treatment of restless legs syndrome. J Acupunct Moxibust 2011;27:7–10. In Chinese.

[9] Moher D Shamseer L Clarke M Preferred reporting items for systematic review and meta-analysis protocols (PRISMA-P) 2015 statement. Syst Rev 2015;4:1. 25554246 10.1186/2046-4053-4-1 PMC4320440

[10] GSDA B MAAB C MAS A Cardiovascular events reported in randomized controlled trials in restless legs syndrome. Sleep Med 2020;65:13–7. 31706187 10.1016/j.sleep.2019.06.022

[11] Bollu P Goyal M Sivaraman M 0802 to examine the effect of gabapentin enacarbil in primary restless legs syndrome patients who are on dopaminergic agents and exhibiting augmentation. Sleep Supplement_1: A305.

[12] Shaikh AG Mehndiratta P Gonzalez C Analog restless legs syndrome rating scale. Eur Neurol 2013;70:195–200. 23969556 10.1159/000351780

[13] Savović J Weeks L Sterne JA Evaluation of the Cochrane Collaboration's tool for assessing the risk of bias in randomized trials: focus groups, online survey, proposed recommendations and their implementation. Syst Rev 2014;3:37. 24731537 10.1186/2046-4053-3-37 PMC4022341

[14] Cumpston M Li T Page MJ Updated guidance for trusted systematic reviews: a new edition of the Cochrane Handbook for Systematic Reviews of Interventions. Cochrane Database Syst Rev 2019;10:D142. 10.1002/14651858.ED000142 PMC10284251 31643080

[15] Chaimani A Higgins JP Mavridis D Graphical tools for network meta-analysis in STATA. Plos One 2013;8:e76654. 24098547 10.1371/journal.pone.0076654 PMC3789683

[16] Stephenson M Fleetwood K Yellowlees A Alternatives to winbugs for network meta–analysis. Value Health 2015;18:A720.

[17] Ades AE Sculpher M Sutton A Bayesian methods for evidence synthesis in cost-effectiveness analysis. Pharmacoeconomics 2006;24:1–9. 10.2165/00019053-200624010-00001 16445299

[18] Lucchetta RC Riveros BS Pontarolo R Systematic review and meta-analysis of the efficacy and safety of amfepramone and mazindol as a monotherapy for the treatment of obese or overweight patients. Clinics (Sao Paulo) 2017;72:317–24. 28591345 10.6061/clinics/2017(05)10 PMC5439101

[19] Salanti G Ades AE Ioannidis JP Graphical methods and numerical summaries for presenting results from multiple-treatment meta-analysis: an overview and tutorial. J Clin Epidemiol 2011;64:163–71. 20688472 10.1016/j.jclinepi.2010.03.016

[20] Dias S Welton NJ Caldwell DM Checking consistency in mixed treatment comparison meta-analysis. Stat Med 2010;29:932–44. 20213715 10.1002/sim.3767

[21] Sturtz S Bender R Unsolved issues of mixed treatment comparison meta-analysis: network size and inconsistency. Res Synth Methods 2012;3:300–11. 26053423 10.1002/jrsm.1057

[22] Song F Clark A Bachmann MO Simulation evaluation of statistical properties of methods for indirect and mixed treatment comparisons. Bmc Med Res Methodol 2012;12:138. 22970794 10.1186/1471-2288-12-138 PMC3524036

[23] Huedo-Medina TB Sánchez-Meca J Marín-Martínez F Assessing heterogeneity in meta-analysis: Q statistic or I2 index? Psychol Methods 2006;11:193–206. 16784338 10.1037/1082-989X.11.2.193

[24] Copas J Shi JQ Meta-analysis, funnel plots and sensitivity analysis. Biostatistics 2000;1:247–62. 12933507 10.1093/biostatistics/1.3.247

[25] Sutton AJ Duval SJ Tweedie RL Empirical assessment of effect of publication bias on meta-analyses. BMJ 2000;320:1574–7. 10845965 10.1136/bmj.320.7249.1574 PMC27401

